# Pathogenic Role of Basic Calcium Phosphate Crystals in Destructive Arthropathies

**DOI:** 10.1371/journal.pone.0057352

**Published:** 2013-02-28

**Authors:** Hang-Korng Ea, Véronique Chobaz, Christelle Nguyen, Sonia Nasi, Peter van Lent, Michel Daudon, Arnaud Dessombz, Dominique Bazin, Geraldine McCarthy, Brigitte Jolles-Haeberli, Annette Ives, Daniel Van Linthoudt, Alexander So, Frédéric Lioté, Nathalie Busso

**Affiliations:** 1 INSERM, UMR-S 606, Hospital Lariboisière,Paris, France; 2 University Paris Diderot (UFR de Médecine), Sorbonne Paris Cité, Paris, France; 3 Department of Musculoskeletal Medicine, Service of Rheumatology, CHUV and University of Lausanne, Lausanne, Switzerland; 4 Department of Rheumatology, Rheumatology Research and Advanced Therapeutics, Radboud University Nijmegen Medical Centre, Nijmegen, The Netherlands; 5 Service des Explorations Fonctionnelles, Hôpital Tenon, AP-HP, Paris, France; 6 Laboratoire de Physique des Solides, Université Paris Sud, Orsay, France; 7 Mater Misericordiae University Hospital, Dublin, Ireland; 8 Service de chirurgie orthopédique et traumatologique de l'appareil moteur, Department of Musculoskeletal Medicine, CHUV and University of Lausanne, Lausanne, Switzerland; National University of Ireland Galway, Ireland

## Abstract

**Background:**

basic calcium phosphate (BCP) crystals are commonly found in osteoarthritis (OA) and are associated with cartilage destruction. BCP crystals induce in vitro catabolic responses with the production of metalloproteases and inflammatory cytokines such as interleukin-1 (IL-1). In vivo, IL-1 production induced by BCP crystals is both dependant and independent of NLRP3 inflammasome. We aimed to clarify 1/ the role of BCP crystals in cartilage destruction and 2/ the role of IL-1 and NLRP3 inflammasome in cartilage degradation related to BCP crystals.

**Methodology/ Principal Findings:**

synovial membranes isolated from OA knees were analysed by alizarin Red and FTIR. Pyrogen free BCP crystals were injected into right knees of WT, NLRP3 -/-, ASC -/-, IL-1α -/- and IL-1β-/- mice and PBS was injected into left knees. To assess the role of IL-1, WT mice were treated by intra-peritoneal injections of anakinra, the IL-1Ra recombinant protein, or PBS. Articular destruction was studied at d4, d17 and d30 assessing synovial inflammation, proteoglycan loss and chondrocyte apoptosis. BCP crystals were frequently found in OA synovial membranes including low grade OA. BCP crystals injected into murine knee joints provoked synovial inflammation characterized by synovial macrophage infiltration that persisted at day 30, cartilage degradation as evidenced by loss of proteoglycan staining by Safranin-O and concomitant expression of VDIPEN epitopes, and increased chondrocyte apoptosis. BCP crystal-induced synovitis was totally independent of IL-1α and IL-1β signalling and no alterations of inflammation were observed in mice deficient for components of the NLRP3-inflammasome, IL-1α or IL-1β. Similarly, treatment with anakinra did not prevent BCP crystal effects. In vitro, BCP crystals elicited enhanced transcription of matrix degrading and pro-inflammatory genes in macrophages.

**Conclusions/ Significance:**

intra-articular BCP crystals can elicit synovial inflammation and cartilage degradation suggesting that BCP crystals have a direct pathogenic role in OA. The effects are independent of IL-1 and NLRP3 inflammasome.

## Introduction

Basic calcium phosphate (BCP) crystals including carbonated-apatite (CA), hydroxyapatite (HA), octacalcium phosphate (OCP), tricalcium phosphate and whitlockite crystals are associated with osteoarthritis (OA), calcific tendinitis, acute arthritis and atherosclerosis (reviewed in [1]). They are identified in the synovial fluid of rapidly destructive OA, as illustrated by the Milwaukee shoulder syndrome [2], and are also highly prevalent in cartilage obtained from affected OA joints at the time of knee joint replacement surgery [3].

Some investigators still consider articular BCP crystals as “innocent bystanders” and/or markers of end-stage OA. Indeed, many cartilage injuries allow subchondral bone mineral to be released into the joint that subsequently deposit onto the cartilage or synovium. However, numerous clinical and experimental reports suggest that cartilage calcification is, in fact, an active process, and can occur in mild OA lesions, and in normal and young cartilage [3], [4], [5]. Furthermore, Sun *et al* have shown that OA meniscal cells upregulate genes involved in the calcification process, that can facilitate crystal formation [6]. Similarly, Fuerst *et al* demonstrated that chondrocytes isolated from OA cartilage generate BCP crystals in conjunction with chondrocyte hypertrophy [3]. This data suggests that both hyaline and fibrous cartilage calcification may be an early and active phenomenon that affects the whole joint and occurs before evidence of cartilage breakdown. Indeed, the guinea pig and STR/Ort murine models of spontaneous OA support this theory, as time-course studies using these animal models demonstrated that, indeed, calcification of the cartilage or menisci as well as the ligaments occur prior to any cartilage breakdown (reviewed in [1]). Finally, in Sprague-Dawley rat, articular cartilage calcification is common and alters cartilage biomechanical properties favouring cartilage destruction [7].

Currently, the mechanisms by which BCP crystals contribute to OA pathogenesis still require further investigation, but it is clear that BCP crystals have multiple effects on articular cells. They are able to induce cellular proliferation, proto-oncogene stimulation, and inflammation related events such as the production and/or activation of cytokines (IL-1 and TNF-β), metalloproteases (MMP), cyclo-oxygenases -1 and -2 and prostaglandin E2 [8], [9], the production of nitric oxide, and the induction of apoptosis in synovial fibroblasts, and articular chondrocytes [10], [11]. They initiate both IL-1β -mediated inflammatory processes through NLRP3 (NACHT-, LRR- and PYD-containing Protein 3)-inflammasome as well as inflammasome-independent pathways [12], [13]. However, despite experimental evidence that suggests the importance of IL-1β in OA pathogenesis [14], clinical studies using IL-1 inhibition in OA treatment have not yielded convincing results [15], [16].

In order to demonstrate the pathogenic role of BCP crystals in the process of destructive arthropathies, we injected HA and OCP crystals, the latter being the most phlogistic one amongst BCP crystals, into murine knee joints. We also explored the mechanisms underlying intra-articular OCP crystals’ effects *in vivo*, taking advantage of mice deficient for different components of the inflammasome, for IL-1α and IL-β, and for Toll-like receptors (TLRs).

## Materials and Methods

### Mice

Female C57BL/6J mice were purchased from Harlan (Horst, The Netherlands). IL-1α-/- and IL-1β-/- mice were a gift from Dr YoichiroIwakura (University of Tokyo, Japan) [17]. Toll-like receptors (TLR) 1, 2, 4, 6 and MyD88 (Myeloid differentiation factor 88) deficient mice were kindly provided by Dr Thierry Roger (Department of infectious diseases, CHUV, Lausanne, Switzerland). ASC-/- (apoptosis-associated speck-like protein containing a CARD) [18] and NLRP3-/- [19] mice were obtained from the late J. Tschopp’s laboratory (Biochemistry Institute, University of Lausanne, Switzerland). All mice were backcrossed onto the C57BL/6 background for at least 9 generations and were compared to WT littermates. Mice were breed under conventional, non-SPF conditions. Mice between 8–12 weeks of age were used for experiments. Animal experiments were performed in strict accordance to the Swiss Federal Regulations. The protocol was approved by the “Service de la consommation et des affaires vétérinaires du Canton de Vaud”, Switzerland (Permit Number: 1908.2). All efforts were made to minimize suffering and minimize the number of mice needed to assess statistical significance and experimental reproducibility.

### Crystal-induced arthritis

Sterile, pyrogen-free HA and OCP crystals were synthesized as previously described [20]. The nature of BCP crystals were checked before and after sterilisation by X-ray diffraction and infrared spectroscopy. X-ray diffraction patterns were recorded with Co-Kalpha (λ = 1.78892Å) using Inel CPS 120 diffractometer operating at 45 kV and 28 mA. Infrared spectra were obtained over the 4000-400 cm^-1^ range, using Nicolet FT-IR 5700 spectrometer with KBr pellet. HA and OCP crystal sizes and Ca/(P+CO3) ratios were determined as previously and were for HA 1.1 ± 0.3 µm and 1.56 and for OCP crystals 1.5 ± 0.5 and 1.33, respectively [20]. Crystals were suspended in sterile PBS and dispersed by brief sonication. All crystals were determined to be endotoxin free (<0.01 EU/10 mg) by Limulus amebocyte cell lysate assay. Crystals (OCP used at 20 or 200 µg in 20 µl endotoxin-free PBS, HA used at 20 µg in 20 µl endotoxin-free PBS) were injected into the right knee joint (i.a.) of mice anaesthetised with 2.5% isoflurane, the left knee joint injected with 20 µl of PBS as a control.

### Anakinra treatment

Anakinra, the recombinant form of IL-1Ra, was injected i.p. twice daily at a dose of 200 µg/mouse for 4 days, the first injection being 30 min prior to intra-articular BCP crystal injection into the knee.

### Isotopic quantification of joint inflammation

Joint inflammation was measured by ^99m^Technetium (Tc) uptake in the knee joint, as previously described [21]. The ratio of Tc uptake in the inflamed arthritic knee versus Tc uptake in the ipsilateral control knee was calculated. A ratio higher than 1:1 indicated joint inflammation.

### Histological examination of knee joints

Mice were sacrificed, the knees dissected, and fixed in 10% buffered formalin for 4 days. Fixed tissues were decalcified in 5% formic acid, dehydrated, and embedded in paraffin. Sagittal sections (6 µm) of the whole knee joint were stained with Safranin-O and counterstained with fast green/iron hematoxylin. Histological sections were graded by two independent observers (VC and NB) unaware of animal genotype or handling. Two different parameters, synovial inflammation and cartilage PG loss were scored on a scale of 0 to 6 in proportion to severity. Von Kossa staining was performed on knees embedded in methyl-methacrylate as described [22].

### Immunohistochemistry

Macrophage and neutrophil (PMN) infiltrates and endothelial cells in knee synovium were detected using anti- Mac-2, anti-MPO or anti-ICAM primary antibodies (all from Sigma-Aldrich, Buchs Switzerland), and visualized using the avidin-biotin-horseradish peroxidase (HRP) complex (Vectastain Elite ABC kit; Vector Laboratories, Burlingame, CA, USA). The color was developed by 3,3′-diaminobenzidine (Sigma-Aldrich, Buchs, Switzerland) containing 0.01% H2O2. Slides were counterstained with Papanicolaou (Merck AG, Dietikon, Switzerland). Apoptotic cells were detected with apopTag Kit (S7100) according to manufacturer’s instructions (Chemicon, Temecula, California, USA). Briefly, DNA fragments labelled in situ by terminal deoxynucleotidyl transferase (TdT) and digoxigenin-nucleotide were detected by anti-digoxigenin antibody conjugated to peroxydase. Negative control sections were performed without TdT. Apoptotic chondrocytes were counted per field (180×140 µm, 3 different fields/mouse) by an observer (VC) who was blinded with regard to mice groups.

MMP-induced neoepitope VDIPEN staining was performed with affinity-purified anti-VDIPEN IgG overnight at 4°C as previously described [23]. Scoring was performed by two independent observers (VC and NB) on an arbitrary scale from 0 to 3 according to the immunostained areas.

### Macrophage stimulation experiments and real-time PCR

C57BL/6 mice were sacrificed and bone marrow cells recovered from tibial and femoral bones. The cells were cultured for 7 days in L929 conditioned media to allow differentiation into macrophages, as described previously [13]. Bone marrow derived macrophages (BMDM) were then stimulated with 500 µg/ml of OCP crystals for 4 hours in RPMI with 1% penicillin/streptomycin (Invitrogen^TM^). RNA was extracted (RNA Clean & Concentrator5-Zymoresearch), reverse transcribed (Superscript II- Invitrogen^TM^), and quantitative Real Time PCR (qRT-PCR) with gene specific primers using the LightCycler480®system (Roche Applied Science) was performed (Table 1). Data was normalized against Tbp and Gapdh references genes, with fold induction of transcripts calculated against the unstimulated control cells.

**Table 1:** Gene specific primers for Real time PCR analysis.

Gene	Forward Primer (5′ 3′)	Reverse Primer (5′ 3′)
Ccl3	CCA AGT CTT CTC AGC GCC AT	TCC GGC TGT AGG AGA AGC AG
Tbp	CTT GAA ATC ATC CCT GCG AG	CGC TTT CAT TAA ATT CTT GAT GGT C
Sdc4	TCT TTG AGA GAA CTG AGG TCT TG	GTC GTA ACT GCC TTC GTC
Sdc1	GTG GCT GTA AAT GTT CCT CC	ACA GAA GGG AAG GAG TAC AT
S100a8	CCA TGC CCT CTA CAA GAA TGA	ATC ACC ATC GCA AGG AAC TC
S100a9	TTA CTT CCC ACA GCC TTT GC	AGG ACC TGG ACA CAA ACC AG
*Rage* Gapdh	ACA TGT GTG TCT GAG GGA AGC CTC ATG ACC ACA GTC CAT GC	AGC TCT GAC CGC AGT GTA AAG CAC ATT GGG GGT AGG AAC AC
Mmp3	ATA CGA GGG CAC GAG GAG	AGA AGT AGA GAA ACC CAA ATG CT
Mmp13	GCA GTT CCA AAG GCT ACA AC	GCT GGG TCA CAC TTC TCT G
Mmp9	AAT AAA GAC GAC ATA GAC GGC A	AAG AGC CCG CAG TAG GG
*Mmp14* Il1a	CAG TAT GGC TAC CTA CCT CC AAA CAC TAT CTC AGC ACC ACT TG	TTG ATC TCA GTC CCA AAC TTA TCC GGT CGG TCT CAC TAC CTG TG
Il1b	CCA CCA ACA AGT GAT ATT CTC CAT G	GTG CCG TCT TTC ATT ACA CAG
Il6	CTG GAC CTC TGC CCT CTG G	TCC ATG GCC ACA ACA ACT GA
Tnfa	CAT CTT CTC AAA ATT CGA GTG ACA A	TGG GAG TAG ACA AGG TAC AAC CC
Adamst4	GCC CGA GTC CCA TTT CCC GC	GCC ATA ACC GTC AGC AGG TAG CG
Adamts5	GAC AGA CCTA CGA TGC CAC CCA GC	ATG AGC GAG AAC ACT GAC CCC AGG
Nos2	ACT ACT ACC AGA TCG AGC C	ACC ACT TTC ACC AAG ACT CTA
Ccl5	TCT CCC TAG AGC TGC CT	TCC TTG AAC CAA CTT CTT CTC TG
Cxcl1	GCC TAT CGC CAA TGA G	CTATGACTTCGGTTTGGG
Cxcl2	ATC CAG AGC TTG AGT GTG ACG C	AAG GCA AAC TTT TTG ACC GC

### OA synovial membrane analysis

OA synovial membranes from 31 patients with different radiographic OA Kellgren-Lawrence scores were collected during knee arthroscopy (scores 0 to 3) or total knee joint replacement (score 4), fixed in 10% buffered formalin for 4 days, dehydrated, and embedded in paraffin. All the patients signed informed consent for obtaining synovial specimens of the operative tissue. The study was approved by the ethics committee of the University Hospital of Lausanne. Sections (6 µm) were stained with alizarin-red. For some OA synovial membranes, consecutive sections were analysed by immunohistochemistry. Macrophages, PMNs and endothelial cells in the synovium were detected using anti-CD68, anti-MPO or CD31 (all from Sigma-Aldrich, Buchs Switzerland) primary antibodies, respectively, and visualized as described in the immunohistochemistry section above. Synovial membrane tissues were also examined by Fourier-transform infrared spectroscopy (FTIR). Mineral phase was evaluated by FT-IR Bruker Vector 22 (BruckerSpectrospin, Wissembourg), according to analytical procedure using the KBr pellet method, as previously described [24], [25].

### Statistical analysis

All values are expressed as the mean ± SEM. Variation between data sets was evaluated using the Student’s t test or one-way ANOVA test, where appropriate, with a 95% confidence interval. Differences were considered statistically significant for a value of p < 0.05. Data was analysed with GraphPad Prism software (GraphPad software).

## Results

### 1. Intra-articular injection of BCP crystals induces synovial inflammation in mice

**Figure 1:**
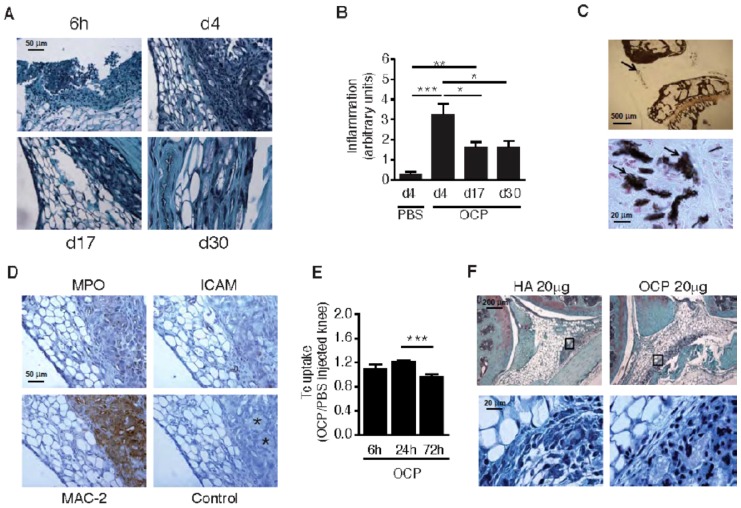
Intra-articular BCP crystals induce synovial inflammation and cartilage proteoglycan loss in mice. OCP crystals (200 µg/20 µl) were injected into right knees of C57BL/6 mice whereas 20 µl PBS was injected into the left knees (A–E). Knees were harvested at different times (day 4, 17 and 30 n = 8 mice per group). Sections were stained with fast green/iron hematoxylin (A) and the degree of inflammation was assessed at the different time points (B). Since the inflammation was very low and similar at all time points in the PBS-injected control knees, only data from PBS-injected knees at day 4 was shown in B. OCP crystal deposition in the synovial membrane was evidenced at day 30 after OCP crystals injection by Von Kossa staining (see arrows) (C). Macrophage, endothelial and PMN cells were detected using antibodies for MAC-2, ICAM, and MPO, respectively, at day 4 after OCP injection (D). Isotype controls allowed the identification of giant cells that had engulfed tissue crystal deposits (*) (D). Ratio of Tc uptake between OCP-injected (n = 8) versus PBS controls was calculated (E). Fast green/iron hematoxylin staining of knees injected with 20 µg/20 µl of HA or OCP crystal at day 4 (F). Results are expressed as mean ± S.E.M with significance being at * p<0.05, ** p<0.01, *** p<0.001.

To assess the role of BCP crystals in cartilage degradation, we performed intra-articular injections of OCP crystals into mouse right knees and an equivalent volume of PBS into the contralateral knee. Joints were evaluated up to 30 days, using immuno/histology methods. We observed a persistent, significant increase in the degree of inflammation (up to day 30) as compared to PBS controls (which showed almost no inflammation at all time points examined), with peak inflammation observed at day 4 (Figure 1A and 1B). OCP crystals were still present in the joints 30 days post-injection, and were predominantly found within the synovial membrane as evidenced by Von Kossa staining (Figure 1C). At 6h, neutrophils predominated the inflammatory infiltrate (results not shown). At later times (d4 onwards), neutrophils were no longer abundant but the membranes showed prominent macrophage infiltration and the presence of occasional multinucleated giant cells with internalized crystals (Figure 1D). Almost no neovascularisation was found, as evidenced by ICAM staining (Figure 1D). We also assessed inflammation by ^99m^Technetium (Tc) scintigraphy. OCP crystal injection induced a small but significant increase in Tc uptake in the knee joints that occurred as an early transient event that peaked at 24 hours, and returned to normal by 72 hours (Figure 1E). Similar histological features of inflammation were also observed with lower doses of OCP and HA crystals (20 µg) into the mouse knee (Figure 1F).

### 2. Intra-articular calcium crystals induced cartilage degradation

**Figure 2:**
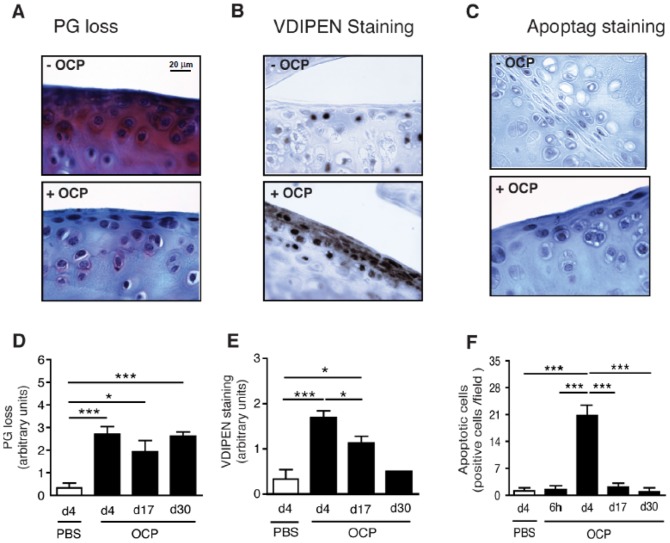
OCP crystals induce cartilage degradation. C57BL/6 mice were injected with OCP crystals (OCP+) or PBS (OCP-). Knees harvested at different times (day 4, 17 and 30 n = 8 mice per group) were assessed for cartilage PGs with Safranin-O (A), aggrecan degradation via VDIPEN immunohistochemistry (B) and apoptosis (C). Since at all time points, data from PBS-injected control knees were similar, only data from PBS-injected knees at day 4 were shown in D, E, and F. Scoring of PG loss and VDIPEN staining was performed on sections, using a scale of 0 to 6 and 0 to 3, respectively (D and E). Apoptotic chondrocytes were counted per field of view (F). Results are expressed as mean ± S.E.M with significance being at * p<0.05, ** p<0.01, *** p<0.001

We evaluated the effects of crystal injection on cartilage integrity at different times following intra-articular injection. PBS injection into the contralateral knee served as a control. OCP crystals induced cartilage proteoglycan loss (PG) as evidenced by loss of Safranin-O staining (Figure 2A, and D) that was already present at day 4 and persisted through to d30. Signs of MMP-mediated aggrecan degradation were also observed by positive VDIPEN staining, that was prominent on d4 and d17, returning to near normal levels at d30 (Figure 2B and E). This was accompanied by chondrocyte apoptosis (Fig 2 C and F), which was maximal at d4. Therefore the sustained PG loss induced by BCP crystals (up to day 30) seemed to be accounted for by both early chondropoptosis (at day 4) and increased MMP activity (at day 4 and 17). However, no typical features of OA such as fissurations or fibrillations of cartilage were found. Taken together, these results indicate that calcium crystals are potent inducers of cartilage degradation as well as synovial inflammation.

### 3. Articular effects of BCP crystals are independent of IL-1 secretion or signalling

Previous data suggested that IL-1 might play a crucial role in the pathogenesis of OA, and that inflammasome activation leading to IL-1β secretion may be important. We investigated the contributions of IL-1 signalling to synovial inflammation and cartilage degradation in this model, using mice deficient for components of the inflammasome, IL-1α and IL-1β. We also tested if IL-1 inhibition using IL-1ra (anakinra) modified the joint pathology.

**Figure 3:**
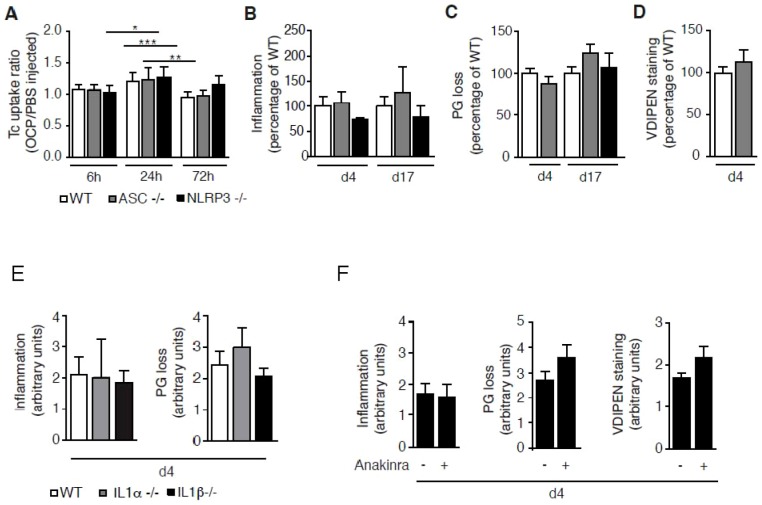
OCP crystal-induced inflammation and cartilage degradation is NLRP3 inflammasome- and IL-1 independent. WT (n = 6), ASC-/- (n = 4), NLRP3-/- (n = 6), IL-1α-/- (n = 5) and IL-1β-/- (n = 6) mice were injected i.a. with OCP crystals (200 µg in 20 µl) or PBS. In a second set of experiment, anakinra, the recombinant form of IL-1Ra, or PBS were injected for 4 days (7 mice per group), the first injection being 30 min prior to OCP injection into the knee of WT (F). Ratio of isotope uptake into OCP injected knee versus PBS-injected ones was calculated at different time points (A). Synovial inflammation (B, E, F), cartilage PG loss (C, E, F) and VDIPEN immunohistochemistries (D, F) were assessed. Results are expressed as % of scores against WT (B,C,D) or in arbitrary units (E, F), and represent mean ± S.E.M. of at least n = 4 mice per group. For p values, *  =  p<0.05, **  =  p<0.01, ***  =  p<0.001.

Knee joints of NLRP3 and ASC deficient mice injected with BCP crystals had similar inflammation, PG loss and VDIPEN-staining scores compared to WT mice (Figure 3A, B, C, D). These results suggest that the NLRP3-inflammasome pathway of IL-1β production is not necessary in crystal-mediated cartilage destruction. To test if either IL-1α or-β were directly involved, we then injected crystals into knee joints of IL-1β or IL-1α deficient mice or WT mice. We observed no significant reduction of inflammation or cartilage damage in the deficient mice (Figure 3E). Finally, we investigated the effects of IL-1Ra, which blocks the binding of both IL-1α and -β to the IL-1 receptor (IL-1R). Mice were injected twice daily i.p. with recombinant anakinra at 200 µg per mouse for 4 days prior to sacrifice, at day 5. The treatment had no effect on crystal-induced synovial inflammation, PG depletion, or VDIPEN staining (Figure 3F).

### 4. OCP crystals induce the expression of matrix degrading genes by macrophages

**Figure 4:**
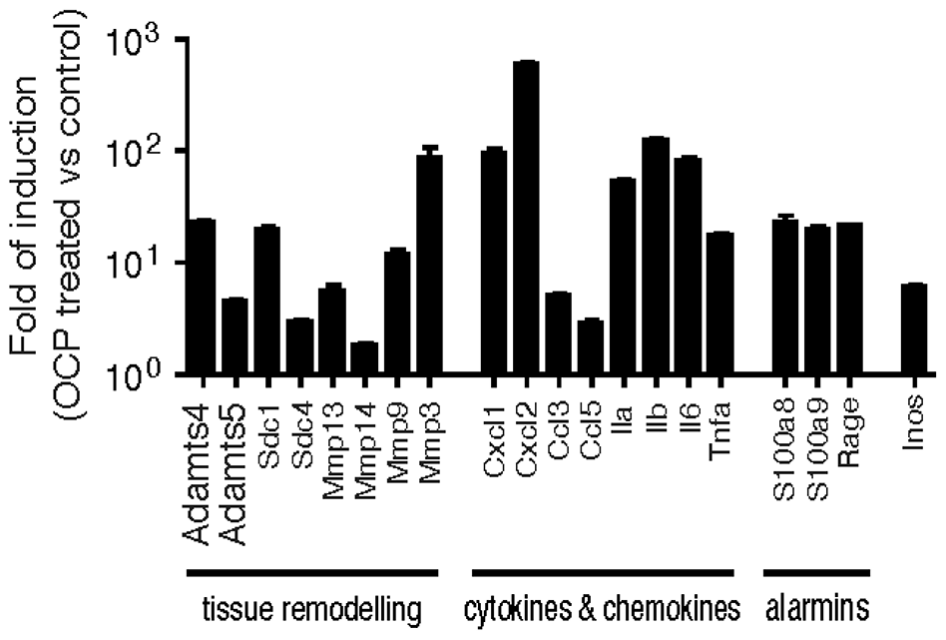
OCP crystals induce macrophage expression of genes involved in inflammation and cartilage degradation. Bone marrow derived macrophages were stimulated *in vitro* with 500 µg/ml of OCP crystals for 4 hours. RNA was extracted, reverse transcribed and qRT-PCR performed using gene specific primers with Tbp, and Gapdh as reference genes. Results are expressed as the fold induction of OCP treated over unstimulated macrophages, using the mean ± S.E.M of triplicate samples.

In view of the predominant macrophage infiltrate within the synovium of OCP injected mice, we hypothesized that crystals induce the expression of macrophage genes that lead to cartilage damage. We stimulated bone marrow derived macrophages from mice with OCP crystals, and analysed by qRT-PCR the expression of inflammatory cytokine and matrix modifying genes (Figure 4).We found dramatically increased expression (>10x compared to control) of *ADAMTS4*, syndecan 1 (*SDC1*), *MMP3* and *9*, *CXCL1* and *CXCL2*, as well as the cytokines *ILA, IL1B, IL6 and TNFA*. The alarmins *S100A8* and *A9* were also upregulated significantly (Figure 4).

### 5. BCP crystals and synovial inflammation in OA patients

**Figure 5:**
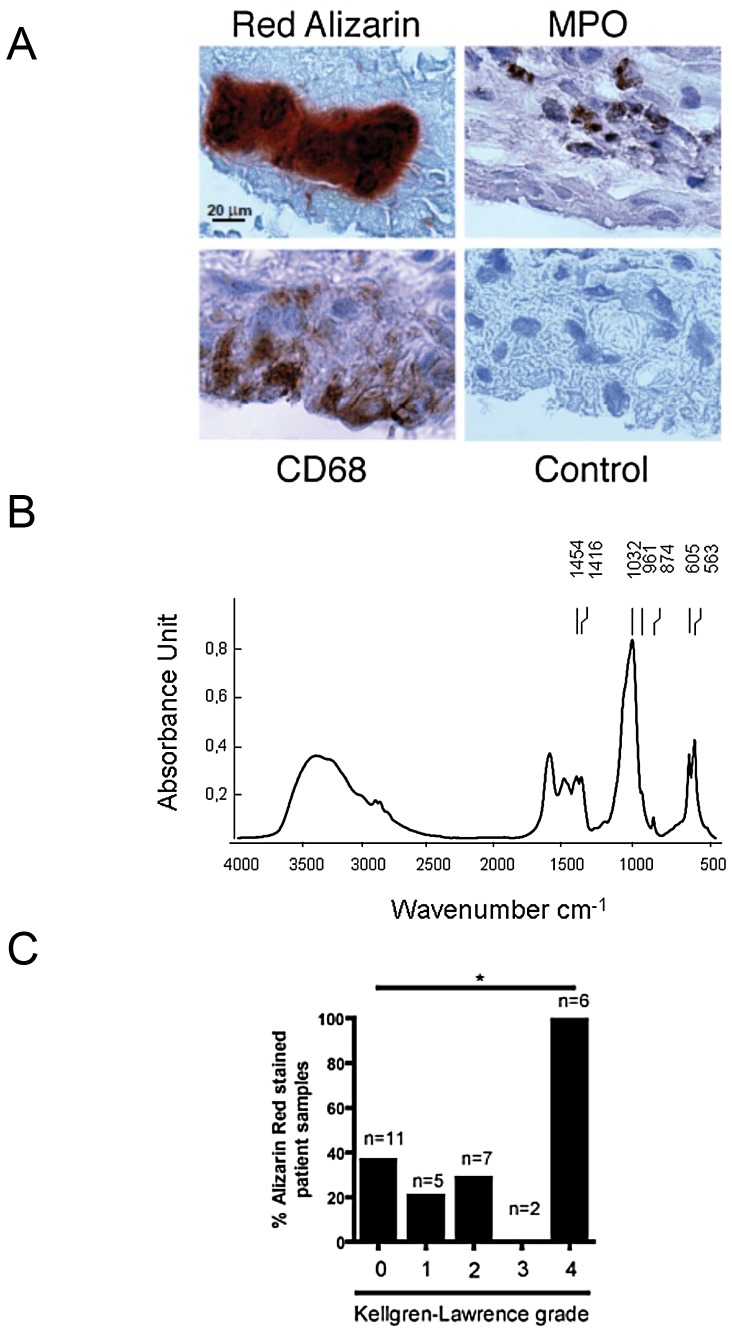
OA synovial membranes contained BCP crystals. Consecutive paraffin sections of OA synoviums were analyzed with alizarin-red staining or by immunohistochemistry using anti-CD68 and anti-MPO primary antibodies (A). Biochemical composition of calcium-containing crystals in OA synovial membranes was assessed by FTIR spectroscopy, displaying a characteristic spectrum of Carbonated apatite (CA), with an absorption band peak at approximately 1035 cm-1 (B). Synovial membranes of different OA grades as assessed by Kellgren Lawrence score were harvested during arthroscopy. FTIR analysis showed the frequent presence of CA crystals in synovium membranes (C).

In parallel to the murine joints evaluation, we analyzed synovial membranes from six OA patients obtained at time of knee joint replacement. All synovial tissues stained positively by alizarin red. In addition, we observed that calcium crystal deposits were surrounded by inflammatory cells, mainly macrophages, as shown by CD68 immunohistochemistry, while neutrophil and endothelial staining was much less abundant (Figure 5A). Analysis with Fourier-transformed infrared spectroscopy (FTIR) clearly showed the presence of BCP crystals, specifically CA (Figure 5B) (17, 18). Thus, the histological findings in human OA synovium were very similar to those observed in the mouse knee joint injected with BCP crystals. Finally, we explored the presence of calcium crystals in a series of synovial membranes harvested during arthroscopy performed in patients with different OA severity stages (Kellgren-Lawrence grades from 0 to 4). Calcium crystals were detected at all stages (except grade 3, were only 2 synovial membranes were analysed). Very interestingly, a significant proportion of synovial membranes from patients not yet diagnosed for OA, were alizarin-red positive (Figure 5C).

## Discussion

The role of calcium phosphate crystals in the pathogenesis of OA is still under debate. Although BCP crystals are highly prevalent in the cartilage of patients presenting for knee and hip surgery in advanced OA [3], [26], there has only been one report of BCP crystals in the synovium [27]. In this study, we showed that calcium-containing microcrystals are found in the 6 synovial membranes in OA at time of joint replacement. We also detected crystals in synovial membrane from low-grade OA (Kellgren-Lawrence score 1–2) and from non affected OA knees (Kellgren-Lawrence score 0). In some cases, we confirmed their composition as BCP using FTIR spectroscopy. The presence of BCP crystals in the synovium suggests that they may be a cause of low-grade inflammation as well as a stimulus for cartilage breakdown. To investigate these effects in more detail, we decided to develop an animal model whereby BCP crystals were injected into the knee joint in vivo. Our findings suggest that BCP crystals do exert a biological effect that is relevant to the pathogenesis of OA.

Following injection of BCP crystals, we observed an early, transient increase in neutrophil recruitment in the synovium at 6 hours post -injection, which was then replaced by a macrophage and multinucleated giant cell rich infiltrate that persisted till day 30. This was observed using different concentrations of OCP crystals as well as a low dose of HA. We also found that the injected crystals persisted within the joint, and were still detectable at up to 30 days when the synovium was stained using von Kossa or alizarin. In the human OA samples, BCP crystal deposits in the synovium co-localized frequently with macrophage infiltration. Similar histological changes have been reported in patients with BCP-associated arthropathy, such as the Milwaukee shoulder syndrome [28] and in canine knee joints that had been injected with calcium pyrophosphate crystals [29]. Additionally, calcium pyrophosphate crystals injected into rabbit knees worsened cartilage OA lesions induced by meniscectomy along with same pattern of synovial inflammation including macrophage infiltration and formation of multinucleated giant cells [30]. Altogether, these results suggest that synovial deposition of calcium-containing crystals leads to synovial inflammation and may enhance joint damage.

Crystal-induced synovitis was accompanied by loss of Safranin-O staining and the appearance of the VDIPEN epitope in cartilage. This epitope, generated by MMPs’ degradation of aggrecan, provides evidence that matrix degradation enzymes are activated within the joint. The cellular sources of these enzymes are multiple. Synovial fibroblasts, synovial macrophages, PMNs and chondrocytes can all produce MMPs, but macrophages are likely to be a major source. We confirmed that transcription of a range of molecules implicated in tissue remodelling was upregulated in BMDM following contact with BCP crystals. The genes include MMPs and ADAMTs. Increased MMP activities have been detected in the synovial fluid of patients with BCP-associated arthropathy [28] and are produced by several cell types.

Other molecules that have been demonstrated to participate in cartilage metabolism or to play a role in the development of experimental OA include the syndecans, the alarmins S100A8 and A9 as well as the inflammatory cytokines IL-1α and -β, TNF-α and IL-6. Their transcription was strongly upregulated by BCP crystals.

As there is a body of evidence that suggests that IL1 plays an important role in OA, and BCP crystals can stimulate IL-1β release by activation of the NLRP3-inflammasome, we expected that mice deficient for components of these pathways would demonstrate an attenuated phenotype following intra-articular injection. Surprisingly, all the deficient strains tested did not demonstrate a reduction in signs of synovial inflammation nor cartilage degradation. We have also tested mice that are deficient in MyD88, TLR-1, -2, -4 and -6 and found no difference in the histological findings (supplement results Fig S1). These results could be explained either by the involvement of cytokines other than IL-1, such as IL-6 and TNF-α, that have been shown to have catabolic effects on cartilage [14] or by pathways of chondrocyte activation that are independent of cytokines. Thus, it was recently shown that mechanical-induced MMP production and cartilage destruction was independent of NLRP3 inflammasome and IL-1 [31]. Monosodium urate (MSU) crystals have been shown to activate Syk kinase following interaction with phospholipids of cell membrane in a receptor independent manner [32], and aluminium crystals as well as MSU activate Syk kinase, PIP3 kinase and prostaglandin synthesis in an inflammasome-independent manner [33]. Very recently, Cunningham et al. showed that BCP crystals induced the production of inflammatory cytokines (IL-1β, IL-1α and TNF-α) by macrophages via Syk and PIP 3 kinase pathways [34]. However, Ng et al. have suggested that Syk was not involved by BCP crystals in dentritic cells [32], [35]. These differences may be secondary to cell types and the characteristics of BCP crystals. Furthers investigations are warranted to clarify this important finding.

In summary, we provide evidence that intra-articular BCP crystals in mouse knees induce synovial inflammation, cartilage degradation and chondrocyte apoptosis and these processes could play a part in the pathogenesis of OA. The findings closely resemble the histological picture seen in patients with moderate and severe OA and suggest that they are of pathologic relevance. The effects observed were independent of the inflammasome-IL-1 pathway that had been described for microcrystal-induced inflammation. We suggest this model may be useful in furthering our understanding of how microcrystals participate in the development or progression of OA.

## Supporting Information

Figure S1 **OCP crystal-induced effects are not mediated by TLRs and MyD88.** Wild-type (WT, n = 10) or knock-out (KO) mice for TLR-1 (n = 8), -2 (n = 12), -4 (n = 10), and -6 (n = 9) and Myd88 (n = 8) mice were injected i.a with 200 µg of OCP crystals into the right knee, the left knee being injected with PBS. Mice were sacrificed at day 4 and histology was performed on the knee joints for inflammation using fast green/iron hematoxylin (A), or PG loss using Safranin O (B). Results are expressed as the mean ± S.E.M with significance being at * p<0.05, ** p<0.01, *** p<0.001.
(TIF)
