# Supplementary figures and images for: Pathogenic Role of Basic Calcium Phosphate Crystals in Destructive Arthropathies
Source: PLoS One. 2013 Feb 28;8(2):e57352. doi: 10.1371/journal.pone.0057352 (PMC3585350; doi:10.1371/journal.pone.0057352)

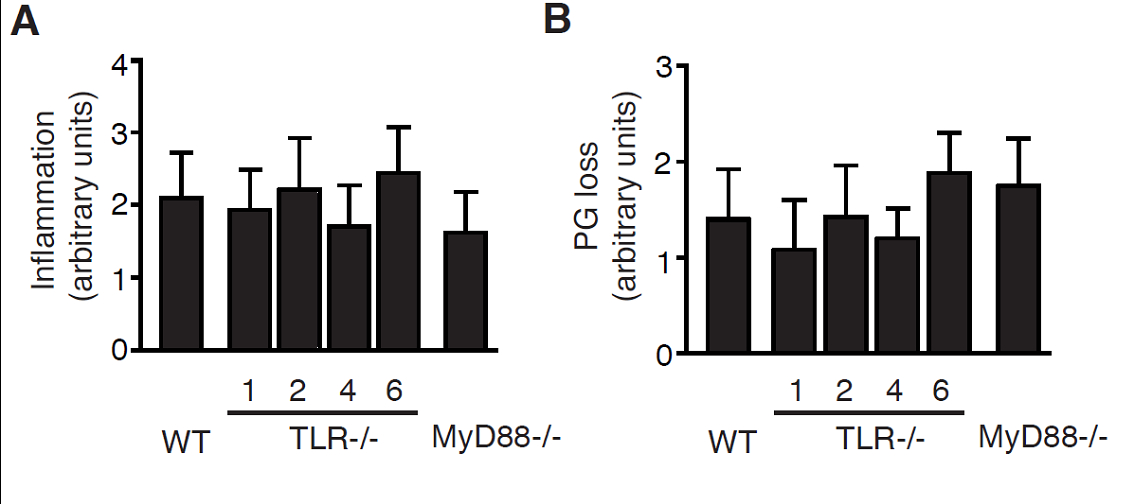

Supplement: Figure S1 — OCP crystal-induced effects are not mediated by TLRs and MyD88. Wild-type (WT, n = 10) or knock-out (KO) mice for TLR-1 (n = 8), -2 (n = 12), -4 (n = 10), and -6 (n = 9) and Myd88 (n = 8) mice were injected i.a with 200 µg of OCP crystals into the right knee, the left knee being injected with PBS. Mice were sacrificed at day 4 and histology was performed on the knee joints for inflammation using fast green/iron hematoxylin (A), or PG loss using Safranin O (B). Results are expressed as the mean ± S.E.M with significance being at * p<0.05, ** p<0.01, *** p<0.001. (TIF) [file pone.0057352.s001.tif]
